# Corrigendum

**DOI:** 10.1002/ehf2.13813

**Published:** 2022-01-17

In the paper by Yazaki *et al*.,^1^ the 9^th^ author's initial was missed.

It should be “Liam M. Heaney” as per the corrected author list below:

Yoshiyuki Yazaki, Kenichi Aizawa, Muhammad Zubair Israr, Keita Negishi, Andrea Salzano, Yuka Saitoh, Natsuka Kimura, Ken Kono, Liam M. Heaney, Shabana Cassambai, Dennis Bernieh, Florence Lai, Yasushi Imai, Kazuomi Kario, Ryozo Nagai, Leong L. Ng, Toru Suzuki.
